# Association of Polymorphism in Pri-microRNAs-371-372-373 with the Occurrence of Hepatocellular Carcinoma in Hepatitis B Virus Infected Patients

**DOI:** 10.1371/journal.pone.0041983

**Published:** 2012-07-27

**Authors:** Min-Sun Kwak, Dong Hyeon Lee, Yuri Cho, Eun-Ju Cho, Jeong-Hoon Lee, Su Jong Yu, Jung-Hwan Yoon, Hyo-Suk Lee, Chung Yong Kim, Jae Youn Cheong, Sung Won Cho, Hyoung Doo Shin, Yoon Jun Kim

**Affiliations:** 1 Department of Internal Medicine and Liver Research Institute, Seoul National University College of Medicine, Seoul, Republic of Korea; 2 Department of Gastroenterology, Ajou University School of medicine, Suwon, Republic of Korea; 3 Department of Life Science, Sogang University, Seoul, Republic of Korea; Drexel University College of Medicine, United States of America

## Abstract

**Background:**

Micro RNAs-371-372-373 (miRNAs-371-373), originating from the same pri-miRNA transcript, are reported to be upregulated in hepatocellular carcinoma (HCC) and to be related to the regulation of hepatitis B virus (HBV) infection. Our study investigated whether pri-miRNAs-371-373 polymorphisms are associated with the risk of HCC occurrence and HBV clearance.

**Methods:**

Genetic variations were identified through direct DNA sequencing using TaqMan assay. Three sequence variants of pri-miRNAs-371-373 were identified. Genetic associations of those with HCC occurrence and HBV clearance among patients with HBV infection were analyzed using logistic regression analyses with adjustment for age and gender (n = 1439).

**Results:**

For the occurrence of HCC, polymorphism rs3859501C>A acted as a protective factor both in chronic carriers (OR = 0.75, *P* = 0.005 in a codominant model; OR = 0.71, *P* = 0.02 in a dominant model; OR = 0.66, *P* = 0.03 in recessive model) and liver cirrhosis patients (OR = 0.69, *P* = 0.001 in a codominant model; OR = 0.60, *P* = 0.003 in a dominant model; OR = 0.63, *P* = 0.03 in a recessive model). The pri-miRNAs-371-373_ht2 [C-A-C] also showed a protective effect on HCC occurrence both in the chronic carrier and liver cirrhosis groups (*P*<0.05 in both). However, there was no significant association between pri-miRNAs-371-373 polymorphisms and HBV clearance.

**Conclusions:**

In conclusion, among chronic carriers and liver cirrhosis patients, the A allele of rs3859501 and the haplotype pri-miRNAs-371-373_ht2 were more protective to HCC than other genotypes and haplotypes. Further studies into the roles of rs3859501 and pri-miRNAs-371-373_ht2 haplotype in hepatocarcinogenesis are needed.

## Introduction

Hepatitis B virus (HBV) infection is a global public health problem as more than 350 million people are suffering from chronic HBV infection worldwide [1]. Patients with HBV infection go through various clinical courses from self-limited infection with spontaneous recovery after hepatitis to chronic HBV infection that may progress to liver cirrhosis (LC) or hepatocellular carcinoma (HCC) [2]. The reasons for this variation in the natural history of HBV infection are not fully understood, but several factors, including virological factors (viral load, genotype, and viral mutation), immunological factors, and host factors like patient age, have been suggested [3], [4], [5]. In addition to these factors, family history is a well-known risk factor for the development of HCC among patients with chronic HBV infection, suggesting the role of a genetic factor in the natural course of HBV [6], [7]. Several previous studies have provided crucial evidence that genetic variations such as polymorphisms of the HDAC10 promoter or polymorphism of TGFBR3 are significantly associated with the risk of HCC or the clearance of HBV [8], [9], [10], [11], [12].

The microRNAs (miRNAs) are a class of short, endogenous, non-coding molecules and are involved in post-transcriptional gene silencing by their binding to specific regions of target mRNA transcripts [13]. MiRNAs are initially transcribed as long, primary miRNA (pri-miRNA) transcripts, which are then processed into hairpin-shaped, precursor miRNAs (pre-miRNAs). The processing of pri-miRNAs is a critical step in miRNA biogenesis, because it defines the miRNA sequences. After processing, pre-miRNAs are subsequently exported to cytoplasm to generate mature miRNAs [14], [15], [16]. It has been reported that miRNAs have critical roles in various physiological processes ranging from cell proliferation, differentiation, and apoptosis to carcinogenesis [17]. Therefore, genetic polymorphisms of pri-, pre-, or mature miRNA genes may alter the expression or target selection of human miRNAs [18], [19], [20]. Relationships between genetic variations in pri-, pre-, and mature miRNAs and various diseases, including cancer susceptibility, have been receiving increasing attention recently.

The miRNAs-372 and -373 (miRNAs-372/373) have been found to be upregulated in HCC or in HCC patients with poor survival [21], [22]. In addition, pre-miRNA-372 was reported to be elevated in LC [23], [24]. Recently, it has been shown that miRNAs-372/373 promote the expression of the HBV through targeting of nuclear factor I/B (NFIB), suggesting a role of miRNAs in the regulation of HBV infection [25].

Therefore, we hypothesized that genetic variations in the pri-miRNA region of miRNA-371-372-373 (pri-miRNAs-371-373) might have an effect on HCC occurrence or on HBV clearance among HBV infected patients. In this study, we investigated the possible association of pri-miRNAs-371-373 polymorphisms with the risk of HCC occurrence and HBV clearance.

## Materials and Methods

### Study patients

A total of 1,439 Korean individuals with either present or past evidence of HBV infection were prospectively enrolled at the outpatient clinic of the liver unit or at the Center for Health Promotion of Seoul National University Hospital and Ajou University Medical Center between January 2001 and August 2003. Diagnosis of chronic HBV carriers were established by repeated seropositivity for the hepatitis B surface antigen (HBsAg; Enzygnost HBsAg 5.0; Dade Behring, Marburg, Germany) over a 6-month period. And spontaneously recovered (SR) individuals were defined as positive anti-HBc (antibody to hepatitis B core antigen; AB-Corek; DiaSorins.r.l., Saluggia, Italy) of the IgG type without HBsAg. Anti-HBs (antibody to hepatitis B surface antigen; Enzygnost Anti-HBs II; Dade Behring) were also evaluated in these patients. The chronic HBV carrier group was assessed further for disease progression to cirrhosis or HCC. All of the patients in the chronic carrier group had undergone regular medical follow-ups and had been evaluated with serum alpha-fetoprotein level, abdominal ultrasonography, and/or a 4-phase spiral liver CT scan more than twice a year to detect early stages of HCC.

Diagnosis of HCC was based on imaging findings of nodules larger than 1 cm showing intense arterial uptake followed by washout of contrast in the venous-delayed phases in 4-phase multidetector CT scan or dynamic contrast enhanced MRI and/or biopsy [26]. Cirrhosis of the liver, on the other hand, was diagnosed pathologically or based on the clinical evidence of portal hypertension such as visible collateral vessels on the abdominal wall, esophageal varices on esophagogastroscopy, palpable splenomegaly, or definite findings of cirrhotic liver or ascites on sonography [27].

Exclusion criteria for the study patients included the following: (i) tested positive for anti-HBs but not for anti-HBc; (ii) tested positive for anti-HCV or anti-HIV (GENEDIA®; Greencross Life Science Corp., Yongin-shi, Korea, HCV®3.2; Dong-A Pharmaceutical Co., Seoul, Korea); (iii) average alcohol consumption of ≥10 g/day or an average number of ≥1 cigarette pack smoked daily assessed through individual interviews; and (iv) incurrence of other types of liver diseases such as autoimmune hepatitis, toxic hepatitis, hemochomatosis, Wilson's disease, non-alcoholic steatohepatitis, primary biliary cirrhosis, or Budd-Chiari syndrome; (v) patients who had a previous history of anti-viral treatment or immunosuppressant. Finally, written consent was secured from the patients prior to conducting this study, and ethical approval was obtained from the Institutional Review Board for Human Research at Seoul National University Hospital and Ajou University Medical Center.

### Genotyping of pri-miRNAs-371-373 genome polymorphisms

Using the Wizard genomic DNA purification kit (Promega, WI, USA), genomic DNA was extracted from patients' peripheral blood samples. Single nucleotide polymorphism (SNP) genotyping was performed using the TaqMan® assay in the ABI prism 7900HT sequence detection system (Applied Biosystems, CA, USA) [28]. Genotyping quality control was performed in 10% of the samples by duplicate checking (rate of concordance in duplicates = 100%). Assay IDs of rs28461391, rs3859501, rs12983273 were ‘C__60779844_10’, ‘C__26894229_10’ and ‘C__1997411_20’, respectively (Applied Biosystems, CA, USA).

### Statistical Analyses

To determine the association of rs28461391, rs3859501, rs12983273 with HCC occurrence and HBV clearance, the odds ratio (OR) and 95% confidence interval (CI) were calculated using logistic analysis adjusted for age (continuous value) and sex (male = 0, female = 1) as covariates to eliminate or reduce any confoundings that might influence the findings. Data was managed and analyzed using the Statistical Analysis System (SAS) version 9.1 (SAS Inc., Cary, NC, USA). Using the PHASE algorithm, version 2.0 [29], haplotypes were then inferred from the genotyped SNPs. Lewontin's D′ (|*D′*|) and the linkage disequilibrium coefficient *r* ^2^ were then examined using the Halploview algorithm [30] to measure the linkage disequilibrium among all pairs of biallelic loci.

## Results

### Pri-miRNA region of miRNA-371,- 372, and 373

**Figure 1:**
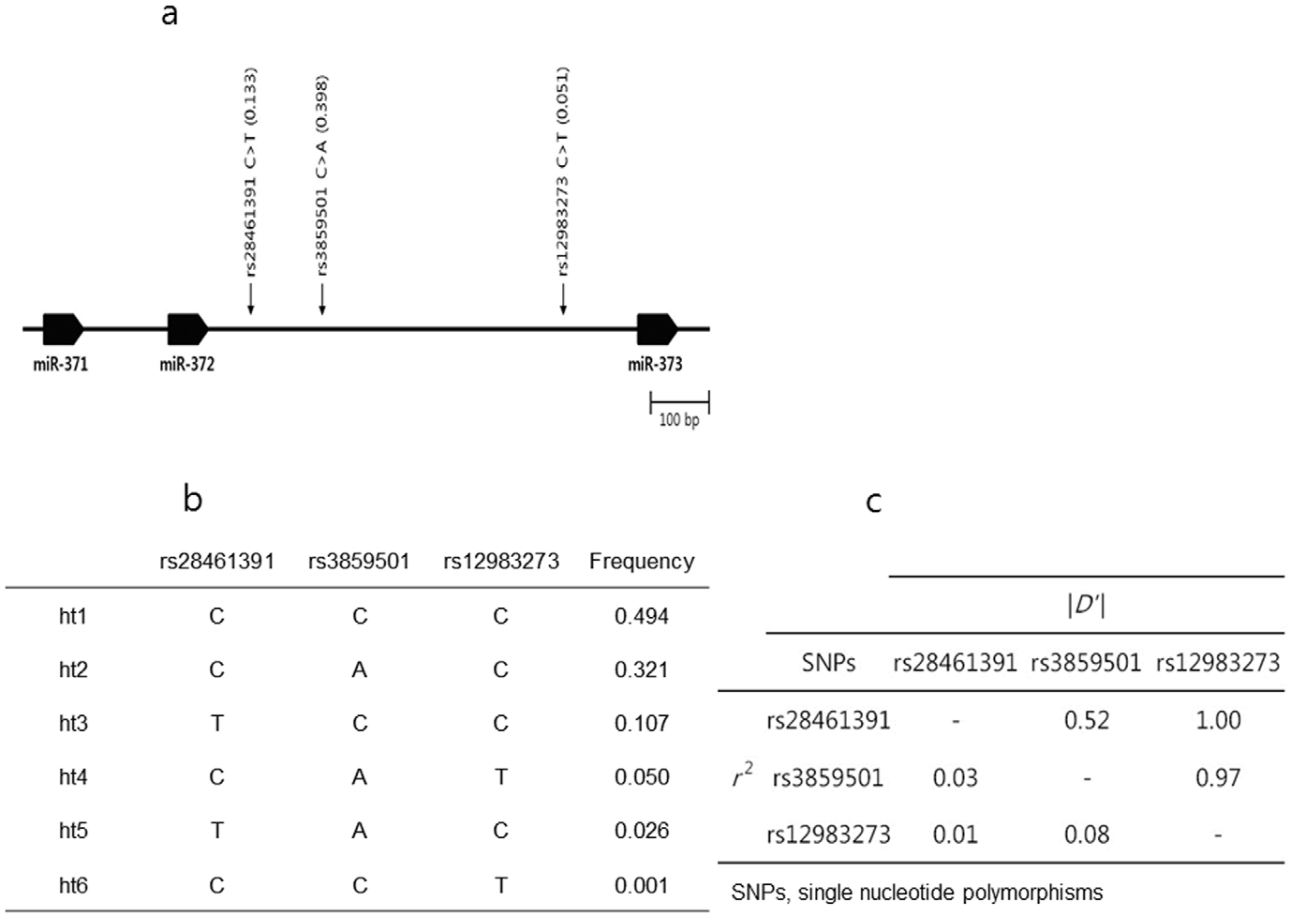
Physical map, haplotypes, and linkage disequilibrium of rs28461391, rs3859501, rs12983273. (a) Schematic gene map and single nucleotide polymorphisms in the pri-miRNA region of miRNA-371-372-373 on chromosome 19q13.42. Black blocks represent miRNA-371, -372, and -373, respectively. The frequency in parentheses was based on the genotyping data (n = 1,439). (b) Haplotypes of rs28461391, rs3859501, rs12983273. (c) Linkage disequilibrium among the three single nucleotide polymorphisms. The upper right-hand and lower left-hand triangles depict the absolute value of standardized disequilibrium coefficient |*D′*| and the correlation coefficient *r* ^2^.

MiRNA 371, 372, and 373 share the same precursor, named pri-miRNA transcribed from chromosome 19q13.42. By direct sequencing, three SNPs were identified in the pri-miRNA region of miRNA 371, 372, and 373. Figure 1a shows schematic gene map and SNPs. Using the PHASE algorithm, 6 haplotypes were inferred from those SNPs. They were ht1(C-C-C), ht2(C-A-C), ht3(T-C-C), ht4(C-A-T), ht5(T-A-C), and ht6(C-C-T) (Figure 1b). The frequency of ht1, most common haplotype, was 0.494. On the other hand, the frequency of ht6 was only 0.001. Linkage disequilibrium was observed among those SNPs (Figure 1c). Absolute value of standardized disequilibrium coefficient |*D′*| between rs28461391 and rs12983273 was 1.00. And the correlation coefficient *r* ^2^ between those SNPs was 0.01. That was the reason, only 6 haplotypes observed, resulted from the combination of 3 SNPs.

### Clinical profiles of the study patients

Total 1,439 Korean subjects having either past or present evidence of HBV infection were enrolled in this study and classified into two subgroups: 404 SR subjects as controls and 1,035 chronic HBV infected patients. Chronic HBV infected patients were composed of 313 chronic hepatitis B subjects, 305 LC subjects, and 417 HCC subjects (Table 1). The mean age of SR was 53.1. In chronic HBV carriers, the mean age varied depending on underlying disease status. Subjects with more progressive disease tended to be older. Not only age, but also male to female ratio, HBeAg, and HBeAb positive rate depended on underlying disease status. Subgroup with more progressive disease had higher male to female ratio, lower positive rate of HBeAg, and higher positive rate of HBeAb.

**Table 1:** Clinical profiles of the study patients.

Clinical profile	Spontaneously recovered	Chronic carrier		
		Hepatocellular carcinoma	Liver cirrhosis	Chronic hepatitis
No. of patients	404	417	305	313
Age [mean (range)]	53.1 (28–81)	57.5 (25–82)	50.8 (22–90)	44.4 (18–79)
Sex (male/female)	272/132	279/48	231/74	238/75
HBeAg (positive rate)	0%	25.7%	29.7%	37.1%
HBeAb (positive rate)	37.7%	65.8%	50.2%	44.9%
HBsAg (positive rate)	0%	100%	100%	100%
HBsAb (positive rate)	100%	0%	0%	0%

### Genotype and minor allele frequency of SNPs

Newly detected polymorphism in the pri-miRNAs-371-373 were rs28461391C>T, rs3859501C>A, and rs12983273C>T in 1,439 Koreans (Table 2). They were all transcribed from chromosome 19 and their coordinates in human genome build 37 were 54291289, 54291411, and 54291832. rs3859501, which had CC, CA, and AA genotypes, had highest minor allele frequency. Its minor allele frequency was 0.398 and its heterozygosity (possibility that its genotype was CA) was 0.479. Deviation of rs3859501 did not follow Hardy-Weinberg equilibrium (HWE). *P* value of deviation from HWE for rs3859501 was 0.415 among SR. Not only rs3859501 but also other SNPs in the pri-miRNAs-371-373 did not follow the HWE. All of them had higher *P* value than 0.05. rs12983273, which had CC, CT, and TT genotypes, had lowest minor allele frequency and that was 0.051. Only one subject had TT genotype at rs12983273 in this study population. Inevitably, its heterozygosity was lowest value among three SNPs.

**Table 2:** Genotype and minor allele frequency of rs28461391, rs3859501, rs12983273 in HBV infected patients (n = 1,439).

rs#	Chromosome	Coordinate*	Genotype (#samples)	Minor allele frequency	Heterozygosity	HWE**
rs28461391	19	54291289	CC (1086)	0.133	0.231	0.177
			CT (323)			
			TT (30)			
rs3859501	19	54291411	CC (519)	0.398	0.479	0.415
			CA (694)			
			AA (225)			
rs12983273	19	54291832	CC (1290)	0.051	0.098	0.772
			CT (146)			
			TT (1)			

* Human genome build 37.

** *P* values of deviation from Hardy-Weinberg Equilibrium among spontaneously recovered group.

### Association analysis of SNPs on the pri-miRNAs-371-373 with risk of liver disease

We analyzed association of SNPs on the pri-miRNAs-371-373 with risk of HCC occurrence and that of HBV clearance (Table 3). rs3859501C>A SNP acted as a protective factor for occurrence of HCC in chronic HBV carrier group (OR = 0.75, 95% CI = 0.61–0.92, *P* = 0.005 in a codominant model; OR = 0.71, 95% CI = 0.53–0.94, *P* = 0.02 in a dominant model; OR = 0.66, 95% CI = 0.45–0.96, *P* = 0.03 in a recessive model). And this SNP also showed protective effect to the occurrence of HCC in LC group (OR = 0.69, 95% CI = 0.55–0.86, *P* = 0.001 in a codominant model; OR = 0.60, 95% CI = 0.43–0.84, *P* = 0.003 in a dominant model; OR = 0.63, 95% CI = 0.41–0.95, *P* = 0.03 in a recessive model). However, HBV clearance rates were not dependent upon SNP genotypes.

**Table 3:** Association analysis of rs28461391, rs3859501, rs12983273 on the pri-miRNA region of miRNAs-371-372-373 with risk of liver disease in HBV infected patients (n = 1,439).

rs#	Minor allele frequency		Codominant		Dominant		Recessive	
	Case	Control	OR (95%CI)	*P*	OR (95%CI)	*P*	OR (95%CI)	*P*
**HCC occurrence**								
HCC occurrence (n = 417) vs. non-occurrence (n = 618) among chronic HBV carriers								
rs28461391	0.122	0.132	0.98(0.74–1.31)	0.91	0.93(0.67–1.29)	0.66	1.61(0.60–4.26)	0.34
rs3859501	0.373	0.414	0.75(0.61–0.92)	**0.005**	0.71(0.53–0.94)	**0.02**	0.66(0.45–0.96)	**0.03**
rs12983273	0.058	0.044	1.22(0.78–1.92)	0.39	1.22(0.78–1.92)	0.39	.	.
ht1 (C-C-C)	0.522	0.484	1.25(1.04–1.52)	**0.02**	1.26(0.92–1.72)	0.15	1.47(1.07–2.01)	**0.02**
ht2 (C-A-C)	0.299	0.341	0.74(0.60–0.91)	**0.005**	0.72(0.55–0.95)	**0.02**	0.57(0.36–0.91)	**0.02**
ht3 (T-C-C)	0.104	0.100	1.09(0.80–1.49)	0.59	1.02(0.73–1.45)	0.89	2.72(0.78–9.57)	0.12
ht4 (C-A-T)	0.056	0.042	1.20(0.76–1.90)	0.43	1.20(0.76–1.90)	0.43	.	.
HCC occurrence (n = 417) vs. non-occurrence (n = 305) among liver cirrhosis patients								
rs28461391	0.122	0.121	1.04(0.75–1.45)	0.82	0.99(0.68–1.43)	0.95	1.84(0.54–6.23)	0.33
rs3859501	0.373	0.444	0.69(0.55–0.86)	**0.001**	0.60(0.43–0.84)	**0.003**	0.63(0.41–0.95)	**0.03**
rs12983273	0.058	0.043	1.27(0.75–2.14)	0.37	1.27(0.75–2.14)	0.37	.	.
ht1 (C-C-C)	0.522	0.466	1.33(1.07–1.65)	**0.01**	1.30(0.91–1.84)	0.15	1.67(1.16–2.42)	**0.006**
ht2 (C-A-C)	0.299	0.370	0.67(0.53–0.85)	**0.0009**	0.65(0.47–0.89)	**0.007**	0.49(0.30–0.81)	**0.005**
ht3 (T-C-C)	0.104	0.090	1.18(0.82–1.70)	0.38	1.13(0.75–1.69)	0.56	2.82(0.58–13.73)	0.20
ht4 (C-A-T)	0.056	0.043	1.24(0.73–2.10)	0.43	1.24(0.73–2.10)	0.43	.	.
**HBV clearance**								
Chronic HBV carriers (n = 1,035) vs. spontaneously recovered (n = 404)								
rs28461391	0.128	0.146	0.86(0.68–1.08)	0.19	0.88(0.67–1.15)	0.34	0.55(0.26–1.15)	0.11
rs3859501	0.398	0.398	1.01(0.85–1.19)	0.95	0.96(0.75–1.22)	0.73	1.10(0.79–1.52)	0.57
rs12983273	0.049	0.057	0.88(0.61–1.27)	0.49	0.90(0.62–1.31)	0.58	.	.
ht1 (C-C-C)	0.499	0.481	1.07(0.91–1.26)	0.44	0.99(0.76–1.28)	0.92	1.22(0.92–1.60)	0.16
ht2 (C-A-C)	0.324	0.316	1.04(0.87–1.24)	0.66	1.02(0.81–1.29)	0.85	1.14(0.77–1.70)	0.52
ht3 (T-C-C)	0.102	0.121	0.82(0.64–1.06)	0.13	0.84(0.63–1.11)	0.23	0.50(0.21–1.21)	0.13
ht4 (C-A-T)	0.048	0.057	0.86(0.59–1.24)	0.41	0.88(0.60–1.28)	0.49	.	.

Minor allele frequencies and *P* values for logistic analyses of three alternative models (co-dominant, dominant, and recessive models) are shown.

Abbreviations: HCC, hepatocellular carcinoma; HBV, hepatitis B virus; OR, odds ratio; CI, confidence interval.

### Association analysis of haplotypes originated from SNPs combination with risk of liver disease

Similar analyses were done in haplotypes from combination of three SNPs. Like rs3859501C>A SNP, one of halplotypes with C-A-C genotypes, ht2 showed protective effect to the occurrence of HCC in chronic HBV carriers (OR = 0.74, 95% CI = 0.60–0.91, *P* = 0.005 in a codominant model; OR = 0.72, 95% CI = 0.55–0.95, *P* = 0.02 in a dominant model; OR = 0.57, 95% CI = 0.36–0.91, *P* = 0.02 in a recessive model). Ht2 had a protective role to the occurrence of HCC in LC group, too (OR = 0.67, 95% CI = 0.53–0.85, *P* = 0.0009 in a codominant model; OR = 0.65, 95% CI = 0.47–0.89, *P* = 0.007 in a dominant model; OR = 0.49, 95% CI = 0.30–0.81, *P* = 0.005 in a recessive model). While ht2 acted as a protective factor for occurrence of HCC, ht1, which consisted of C-C-C genotypes showed susceptible effect to the occurrence of HCC in codominant and recessive models. Six halpotypes did not have any effect on HBV clearance.

## Discussion

This study investigated the genetic associations of the pri-miRNAs-371-373 with HCC occurrence and HBV clearance. We found that the rs3859501 C>A polymorphism and the pri-miRNAs-371-373_ht2 [C-A-C] haplotype were more protective than other polymorphisms and haplotypes to HCC occurrence among chronic HBV carriers and LC patients. However, the pri-miRNAs-371-373 polymorphisms were not associated with HBV clearance.

As host genetic factors can affect the course of HBV infection, many genetic factors have been studied in relation to HCC occurrence and HBV clearance. Recently, miRNAs and their precursors have been studied extensively, by regulating many genes including tumor suppressors and oncogenes, as factors related to many tumors [31], [32]. Aberrant expression and structural alteration of miRNAs are thought to be involved in tumorigenesis and cancer development, including that of HCC [33]. It has been suggested that the presence of SNPs in pri- or pre-miRNAs can alter miRNA processing, expression, and/or binding to target mRNA and may represent another type of genetic variability that can contribute to the development of human cancers [31], [33].

This study showed that a pri-miRNAs-371-373 polymorphism (rs3859501 C>A) was more protective to HCC occurrence among patients with HBV infection. The pri-miRNA polymorphism can affect the expression level of miRNAs or it can affect the function or processing of miRNAs. It has already been reported that miRNA-372 is overexpressed in HCC [21], [22]. Therefore, it may be possible that a polymorphism of pri-miRNAs-371-373 affects the expression level of miRNAs-371-373 clusters; though, further study should be undertaken. According to previous reports, the miRNAs-371-373 cluster is known to be frequently deregulated in other human tumors, such as testicular germ cell tumors [34], hepatoblastoma [35], and colorectal cancer [36]. The diverse action mechanisms of the miRNAs-371-373 cluster on various cancer occurrence have been suggested; for example, collaboration with oncogenic *Ras* and neutralization of p53-mediated cyclin-dependent kinase inhibition [34], promotion of tumor invasion and metastasis by suppression of CD 44 [37], or modulation of Wnt/β-catenin-signaling pathways [38]. These are also known molecular signals in HCC [39], [40], therefore these signals can be possible mechanisms for the effects of pri-miRNAs-371-373 polymorphisms on HCC occurrence.

It has been reported that several miRNAs, such as miRNA-122 are related to the clearance or replication of HBV through interaction with HBV and host miRNAs [41], [42]. The miRNAs-372/373 are transcribed from genomic locus 19q13.41, which is near the genomic site FRA 19A, a known hotspot for the integration of HBV DNA [43]. Therefore, we postulated that pri-miRNAs-371-373 polymorphisms could be associated with the HBV infection. However, no association was found between these three polymorphisms and the resolution of HBV infection in this study. Previous study also showed that miRNAs-372/373 expression was not correlated with hepatic HBV DNA levels, which reflect levels of viral replication [13].

Since polymorphisms may have different effects in different populations and considering that samples in this study were from a homogeneous population, replication studies in HBV infected cohorts from other ethnic groups are recommended. The mechanism relating polymorphism of pri-miRNAs-371-373 to HCC occurrence was not determined in this study and further study may provide useful information about HCC pathogenesis in HBV patients. HBV DNA levels have been regarded as one of factors associated with HBV clearance; however, HBV DNA levels were found to fluctuate during follow-up in the majority of our HBV cohort. Here, therefore, the logistic models for HBV clearance were not adjusted by HBV DNA levels, though other factors, including age, sex were controlled. Also, we controlled previous antiviral agent use and viral genotype by including only the patients without previous antiviral agent use and enrolling only the Korean patients in whom almost all were infected by genotype C HBV [44].

This is the first study to show the effects of pri-miRNAs-371-373 polymorphisms on HCC occurrence and HBV clearance. Our study demonstrated that one pri-miRNAs-371-373 polymorphism (rs3859501 C>A) and the pri-miRNAs-371-373_ht2 [C-A-C] haplotype were more protective to HCC occurrence in patients with HBV infection. However, the polymorphism of pri-miRNAs-371-373 was not associated with spontaneous HBV clearance.
